# Breath Hydrogen Produced by Ingestion of Commercial Hydrogen Water and Milk

**DOI:** 10.4137/bmi.s2209

**Published:** 2009-02-09

**Authors:** Akito Shimouchi, Kazutoshi Nose, Makoto Yamaguchi, Hiroshi Ishiguro, Takaharu Kondo

**Affiliations:** 1 Department of Etiology and Pathogenesis, National Cardiovascular Center Research Institute, Japan; 2 Research Center of Health, Physical Fitness and Sports, Nagoya University, Japan

**Keywords:** breath hydrogen, hydrogen water, milk, colonic fermentation

## Abstract

**Objective:**

To compare how and to what extent ingestion of hydrogen water and milk increase breath hydrogen in adults.

**Methods:**

Five subjects without specific diseases, ingested distilled or hydrogen water and milk as a reference material that could increase breath hydrogen. Their end-alveolar breath hydrogen was measured.

**Results:**

Ingestion of hydrogen water rapidly increased breath hydrogen to the maximal level of approximately 40 ppm 10–15 min after ingestion and thereafter rapidly decreased to the baseline level, whereas ingestion of the same amount of distilled water did not change breath hydrogen (p < 0.001). Ingestion of hydrogen water increased both hydrogen peaks and the area under the curve (AUC) of breath hydrogen in a dose-dependent manner. Ingestion of milk showed a delayed and sustained increase of breath hydrogen in subjects with milk intolerance for up to 540 min. Ingestion of hydrogen water produced breath hydrogen at AUC levels of 2 to 9 ppm hour, whereas milk increased breath hydrogen to AUC levels of 164 ppm hour for 540 min after drinking.

**Conclusion:**

Hydrogen water caused a rapid increase in breath hydrogen in a dose-dependent manner; however, the rise in breath hydrogen was not sustained compared with milk.

## Introduction

It is widely accepted that breath hydrogen reflects carbohydrate fermentation in the colon.^1^–^4^ When unabsorbed carbohydrate enters the colon, it is rapidly fermented to produce short chain fatty acids by anaerobic colonic bacteria and liberates carbon dioxide, hydrogen, and in some people, methane. Analysis of breath hydrogen measures the small bowel transit time,^5^ colonic fermentation, abnormal fermentation, galactose and/or lactose intolerance,^6^ and sometimes irritable bowel syndrome.^4^ Thus, breath hydrogen has been recognized as “a negative breath marker”.

Recent studies revealed that hydrogen gas plays an important role in inactivating oxidative stresses such as hydroxyl radicals in animal models of cerebral infarction,^7^ hepatic injury^8^ and myocardial ischemia-reperfusion injury.^9^ Very recently, it has been reported that continuous consumption of hydrogen water reduces oxidative stress in the brain, and prevents stress-induced decline in learning and memory caused by chronic physical restraint.^10^ These reports suggest that breath hydrogen, originating mostly from colonic fermentation, may be an anti-oxidative marker, provided that molecular hydrogen, produced as a by-product of colonic fermentation, acts as a reducing agent.^11^

Since hydrogen solubility in water at 25 °C is 0.0176 vol gas STP/vol liquid at 1 atm partial pressure,^12^ which is lower than that of oxygen and carbon dioxide and higher than that of nitrogen,^13^ it is easily calculated that hydrogen-saturated water contains 0.79 mM of hydrogen at 25 °C. Countless commercial hydrogen water products are marketed mostly in Japan as health-oriented water. However, clinical evidence regarding hydrogen water is slight, except for a recent clinical trial for type 2 diabetes mellitus,^14^ which did not report detailed alterations of breath hydrogen after drinking it and, a comparison was not made with reference foods or changes of breath hydrogen after everyday diet.

In the present study, in order to examine whether ingestion of commercial hydrogen water increased breath hydrogen and the duration for which it was maintained, the effects of hydrogen water on breath hydrogen was examined. The effect of milk ingestion on breath hydrogen was also examined for comparison as milk increases breath hydrogen.^15^

## Materials and Methods

### Materials

Commercial hydrogen water was purchased at the general consumer level and fresh hydrogen water (0.6 mM) was obtained from Blue Mercury Inc. (Tokyo, Japan). Hydrogen water was made by dissolving hydrogen into water under high pressure, as described by Nagata et al.^10^ Hydrogen concentration was analyzed for each experiment and ranged from 0.21 to 0.58 mM. Hydrogen water contains sodium 1.4 mg/dl; calcium 1.3 mg/dl; potassium 0.4 mg/dl; and magnesium 1.1 mg/dl without protein, carbohydrates and lipids (total energy: 0 kcal). Commercial milk was purchased from Morinaga Milk Industry Co. Ltd. (Tokyo, Japan). Milk contains protein 3.3 g/dl; lipid 3.8 g/dl; carbohydrates 4.75 g/dl; sodium 42.5 mg/dl; and calcium 113.5 mg/dl (total energy of 66.5 kcal/dl). Because cold stimulation^16^ and cold milk^17^ enhance colonic motility and a rise in breath hydrogen, room temperature and test items for ingestion were kept at 25 °C.

### Subjects

Five healthy adult volunteers (two men and three women: mean age 29 ± 14 years) participated in this study. They did not have any specific diseases based on detailed questionnaires and examination by physicians. The subjects randomly volunteered to be assigned to consecutive studies from experiment one to three. All the subjects eventually showed lactose deficiency as described below, possibly due to the high incidence of lactose deficiency in Japanese people.^18^

### Experimental protocol

The subjects refrained from food, supplements and drugs, except water, for at least 15 hours before the experiments. On the day of the experiment, the subjects rested in the sitting position for at least 30 min and then drank 300 ml of milk, hydrogen water or distilled water within one min. Immediately after drinking, the subjects rinsed their oral cavity with 50 ml of distilled water. End-alveolar breath was obtained in a breath sampling bag (300 ml volume, Otsuka Pharm. Co. Ltd, Tokyo, Japan) every 5 min from 30 min before drinking until the end of each experiment as described below. All subjects underwent training to obtain end-alveolar breath before these experiments.

#### Experiment 1

For the dose-response experiment, hydrogen water for general consumers was used within one week after shipping. Hydrogen concentration was 0.40 ± 0.07 mM (N = 18). Three subjects had hydrogen water in volumes of 100, 200 and 300 ml every hour, with two repetitions.

#### Experiment 2

To confirm that breath hydrogen originates from hydrogen water, five subjects had 300 ml of distilled water and then changes in breath hydrogen were observed for 90 min. After an hour , the same subject ingested hydrogen water (0.4 mM).

#### Experiment 3

To compare the hydrogen-producing capability of hydrogen water and milk, four subjects had 300 ml of milk and their breath hydrogen was monitored for up to 540 min. On separate experimental days, the same two subjects drank 300 ml of hydrogen water and repeated the same protocols at three different concentration levels (0.21, 0.41 and 0.58 mM) of hydrogen water.

### Breath sampling and analysis

The breath was immediately transferred to a gas-tight glass syringe and 1 ml was injected into the gas chromatograph with a semiconductor detector (TRIlyzer mBA–3000, Taiyo Ltd, Osaka, Japan) to measure breath hydrogen. Breath concentrations of each gas were calculated by subtracting ambient air collected in the same sampling bag. Hydrogen concentration of the milk, distilled or hydrogen water at 25 °C were measured for each subject by head-space gas sampling in a sealed glass vial with a small amount of the materials. Analysis was performed using the same sensor as mentioned above.

### Statistics

Statistical values are expressed as the mean ± standard deviations of the mean. Statistical analysis was performed by a paired t-test or two-way analysis of variance and significance was accepted at p < 0.05.

## Results

### Experiment 1

**Figure 1:**
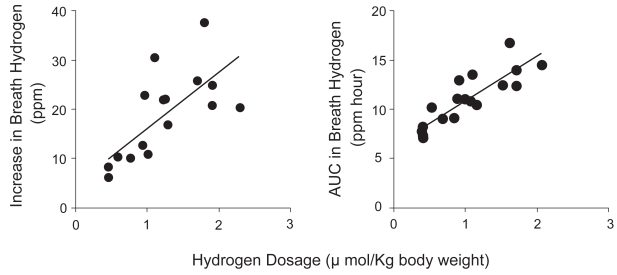
Increase in breath hydrogen and the area under the curve (AUC) of breath hydrogen for 60 min after ingestion were significantly correlated to hydrogen dosage (r = 0.735, p = 0.001; r = 0.855, p < 0.001, respectively). The data were obtained from 3 subjects in duplication by ingestion of 100, 200 and 300 ml of hydrogen water. Hydrogen concentrations of ingestion water for each subject were measured simultaneously.

Hydrogen water significantly increased both hydrogen peak (r = 0.735, p = 0.001) and the area under the curve (AUC) of breath hydrogen (r = 0.855, p < 0.001) in a dose-dependent manner (Fig. 1).

### Experiment 2

**Figure 2:**
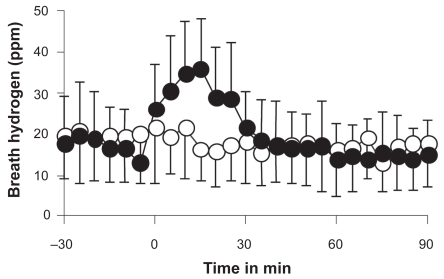
Changes in breath hydrogen after ingestion of distilled water (300 ml, open circle) and hydrogen water (300 ml, 0.4 mM hydrogen, closed circle) in 5 subjects. The difference between the two groups is significant (p < 0.001, two-way analysis of variance).

Effects of distilled and hydrogen water on breath hydrogen were compared. Ingestion of hydrogen water (0.4 mM) rapidly increased breath hydrogen to the maximal level of 36 ppm 15 min after ingestion and thereafter rapidly decreased to the baseline level, whereas the same amount of distilled water ingestion did not change breath hydrogen (ANOVA, p < 0.001, Fig. 2).

### Experiment 3

**Figure 3:**
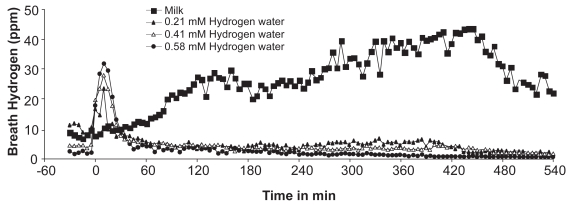
Time course of breath hydrogen by milk (N = 4) and hydrogen water (N = 2 for 0.21, 0.41 and 0.58 mM hydrogen) for 540 min.

Effects of hydrogen water and milk on breath hydrogen for up to 540 min are shown in Figure 3. Ingestion of milk exhibited a delayed and sustained increase of breath hydrogen in subjects with hypolactasia for up to 540 min, during which subjects complained of few bowel symptoms, except normal evacuation in one subject, while those who had hydrogen water showed only an initial increase in breath hydrogen. (Fig. 3). To assess hydrogen production, AUC of breath hydrogen for 540 min after ingestion, subtracting the baseline breath hydrogen, was calculated. Hydrogen water produced breath hydrogen at AUC levels of 2 to 9 ppm hour, whereas milk significantly increased breath hydrogen to AUC levels of 164 ppm hour for 540 min after drinking.

## Discussion

This study confirmed that ingestion of hydrogen water rapidly increased breath hydrogen in a mean time of 10 to 15 min from ingestion to peak breath hydrogen and that breath hydrogen increased in a dose-dependent manner. The same amount of distilled water at 25 °C did not cause a rise in breath hydrogen by the so-called gastroileal reflex;^19^,^20^ therefore, it was concluded that a rapid rise in breath hydrogen originates from hydrogen water and that acute ingestion of hydrogen water rapidly increased hydrogen partial pressure in the upper alimentary tract, and diffused into the sub-mucosal alimentary vessels, and was finally exhaled.

From experiment one, it was estimated that after ingestion of hydrogen water, 72% of hydrogen molecule was exhaled in breath, provided that the minute volume ventilation of the subjects with a mean body weight of 72 kg was 6 L/min and that hydrogen concentrations in the end-tidal breath could be applied to the ventilation volume. In the preliminary experiment, it was confirmed that the loss of hydrogen poured into the cup during the first 3 min was 2%–5% (data not shown). Furthermore, in the recent experiment, it was estimated that after ingestion of hydrogen water, approximately 0.1% of hydrogen was released from the whole body surface and the amount was considerably negligible, which will be reported elsewhere (Nose K, Shimouchi A, et al). On the other hand, it is known that hydrogen produced by colonic fermentation is partially consumed by bacterial flora in the colon.^21^ However, breath hydrogen release was almost finished 60 min after ingestion of hydrogen water, possibly because it was unable to reach colon. Therefore, it was considered that at least 20% of ingested hydrogen was consumed in the body and that the main hydrogen consumption occurred in the liver rather than by the colonic bacteria or other organs such as the brain.

The present study supports the conclusion that ingestion of commercial hydrogen definitely increases hydrogen concentration in the body; however, the rise in breath hydrogen was transient and the hydrogen-producing capability of hydrogen water was less than that of milk in subjects with hypolactasia. Since a higher increase in breath hydrogen was expected, fasted subjects had test items at the rate of 300 ml/60 sec, i.e. 5 ml/sec. However, the rise did not reach the reported level of 55 ppm by ingestion of 300 ml of hydrogen-rich water by a recent clinical trial of Kajiyama et al.^14^ Unfortunately, they did not describe the details of the ingestion methods and hydrogen concentrations. Their patients, who had type 2 diabetes mellitus had 900 ml of hydrogen-rich water per day for 8 weeks; however, their breath hydrogen levels were not reported. The discrepancy may be because the rise in breath hydrogen depended on body weight, the rate of drinking, and preconditioning of baseline levels that are seemingly affected by previous diet or fasting.

On the other hand, Nagata et al.^10^ reported that in mice that were given hydrogen water at hydrogen concentration levels of 0.4–0.6 mM ad libitum, there were differences in the arteriovenous hydrogen concentrations in the blood, and concluded that hydrogen was consumed in the brain of the animals. Although continuous intake of hydrogen water may be impractical in the clinical setting, the new finding showed a new direction for therapies for acute ischemic and reperfusion injuries and prevention;^7^–^9^ however, it should be remembered that hydrogen concentration in the body strongly depends on colonic fermentation and the food consumed everyday. Therefore, further clinical studies are needed to clarify whether exogenous hydrogen, such as hydrogen inhalation or water, could contribute to the increase of hydrogen in the body, which is presumably represented by breath hydrogen.

The same group revealed that hydrogen gas plays an important role in inactivating oxidative stresses such as hydroxyl radicals.^7^–^9^ Their recent reports gave new insight in the roles of hydrogen produced in the body, mostly by colonic fermentation. As a favorable outcome, younger women had higher concentrations of breath hydrogen at baseline levels and in their response to food, both at initial and secondary rises of breath hydrogen, than older women;^22^ therefore, it is of interest that hydrogen produced in the body, to some extent if any, also plays a role in the inactivation of oxidative stress, as low levels of inhaled hydrogen and hydrogen water ad libitum did in rats.

Ingestion of milk in subjects with hypolactasia caused a marked and sustained rise in breath hydrogen, as shown in the present and previous studies.^15^ Approximately 70% of the world population has hypolactasia^23^ and about 90% of Japanese adults are lactase-deficient.^18^ Epidemiological studies suggested that milk and dairy decrease the risks of mortality from cerebrovascular disease or stroke.^24^–^26^ These results have often been attributed to decreases in arterial blood pressure by calcium uptake^27^ or to the reduction of platelet aggregation by short and medium chain fatty acids,^28^ of which the former is produced partially by colonic fermentation and both of which are abundant in milk and dairy food.

This study was performed under special conditions, refraining from unabsorbable foods before the experimental days and eating and drinking nothing except water and the test items. However, in everyday life, breath hydrogen levels fluctuate markedly, ranging from several ppm to 50 ppm or more,^29^ which is enhanced by exercise^30^ and physical and/or mental stresses^16^ in combination with colonic fermentation. Besides milk, breath hydrogen by colonic fermentation is produced by numerous unabsorbable foods, such as commercial dietary fiber,^31^ soybean flour,^32^ and inulin and oligofructose in a number of vegetables, fruits and whole grains.^33^ Thus, hydrogen production appears to be caused naturally by everyday diet, provided there is normal bacterial flora and colonic function. Therefore, this study was conducted to see if additional intake of the commercial preparation of hydrogen water is beneficial for people without specific diseases.

## Conclusion

Hydrogen water caused a rapid increase in breath hydrogen in a dose-dependent manner; however, the rise in breath hydrogen was not sustained compared with the same amount of milk in subjects with milk intolerance.

## References

[1] Levitt MD Ingelfinger FJ Hydrogen and methane production in man Ann N Y Acad Sci 1968 150 75 81 5238616 10.1111/j.1749-6632.1968.tb19033.x

[2] Levitt MD Production and excretion of hydrogen gas in man N Engl J Med 1969 281 122 7 5790483 10.1056/NEJM196907172810303

[3] Levitt MD Donaldson RM Use of respiratory hydrogen (H2) excretion to detect carbohydrate malabsorption J Lab Clin Med 1970 75 937 45 5421079

[4] Simrén M Stotzer PO Use and abuse of hydrogen breath tests Gut 2006 55 297 303 16474100 10.1136/gut.2005.075127 PMC1856094

[5] Kamm MA Leonnard-Jones JE Gastrointestinal transit time Pathophysiology and pharmacology Petersfield Wrightson Biormedical Publishing Ltd 1991

[6] Swagerty DL Jr Walling AD Klein RM Lactose intolerance Am Fam Physician 2002 65 1845 50 12018807

[7] Ohsawa I Ishikawa M Takahashi K Hydrogen acts as a therapeutic antioxidant by selectively reducing cytotoxic oxygen radicals Nat Med 2007 13 688 94 17486089 10.1038/nm1577

[8] Fukuda K Asoh S Ishikawa M Yamamoto Y Ohsawa I Ohta S Inhalation of hydrogen gas suppresses hepatic injury caused by ischemia/reperfusion through reducing oxidative stress Biochem Biophys Res Commun 2007 361 670 4 17673169 10.1016/j.bbrc.2007.07.088

[9] Hayashida K Sano M Ohsawa I Inhalation of hydrogen gas reduces infarct size in the rat model of myocardial ischemia-reperfusion injury Biochem Biophys Res Commun 2008 373 30 5 18541148 10.1016/j.bbrc.2008.05.165

[10] Nagata K Nakashima-Kamimura N Mikami T Ohsawa I Ohta S Consumption of Molecular Hydrogen Prevents the Stress-Induced Impairments in Hippocampus-Dependent Learning Tasks during Chronic Physical Restraint in Mice Neuropsychopharmacology 2009 34 501 8 18563058 10.1038/npp.2008.95

[11] Neale RJ Dietary fibre and health: the role of hydrogen production Med Hypotheses 1988 27 85 7 2849711 10.1016/0306-9877(88)90091-6

[12] Young CL Hydrogen and Deuterium IUPAC Solubility Data Series 5/6 Oxford, England Pergamon Press 1981

[13] Radford EP Jr Fenn OF Rahn H The physics of gases Handbook of Physiology-Respiration I American Physiological Society Washington DC 1964 125 52

[14] Kajiyama S Hasegawa G Asano M Supplimentation of hydrogen-rich water improves lipid and glucose metabolism in patients with type 2 diabetes or impaired glucose tolerance Nutrition Research 2008 28 137 43 19083400 10.1016/j.nutres.2008.01.008

[15] Kondo T Liu F Toda Y Milk is a useful test meal for measurement of small bowel transit time J Gastroenterol 1994 29 715 20 7874265 10.1007/BF02349276

[16] O’Brien JD Thompson DG Burnham WR Holly J Walker E Action of centrally mediated autonomic stimulation on human upper gastrointestinal transit: a comparative study of two stimuli Gut 1987 28 960 9 3666564 10.1136/gut.28.8.960 PMC1433154

[17] Kagaya M Iwata N Toda Y Mitsui T Nakae Y Kondo T Cold milk accelerates orocecal transit time during the luteal phase but not the follicular phase in women Nagoya J Med Sci 1999 62 57 62 10504828

[18] Nose O Iida Y Kai H Harada T Ogawa M Yabuuchi H Breath hydrogen test for detecting lactose malabsorption in infants and children. Prevalence of lactose malabsorption in Japanese children and adults Arch Dis Child 1979 54 436 440 475426 10.1136/adc.54.6.436 PMC1545434

[19] Hertz AF The ileocaecal sphinctor J Phyiol 1913 47 54 6

[20] Douglas DM Mann FC The Activity of the lower part of the ileum of the dog in relation to the ingestion of food Am J Dig Dis 1939 6 434 9

[21] Levitt MD Bond JH Levitt DG Johnson LR Gastrointestinal Gas Physiology of the Gastrointestinal Tracts New York Raven Press 1981 1301 15

[22] Kagaya M Iwata N Toda Y Nakae Y Kondo T Small bowel transit time and colonic fermentation in young and elderly women J Gastroenterol 1997 32 453 6 9250890 10.1007/BF02934082

[23] Lomer MC Parkes GC Sanderson JD Review article: lactose intolerance in clinical practice—myths and realities Aliment Pharmacol Ther 2008 27 93 103 17956597 10.1111/j.1365-2036.2007.03557.x

[24] Kinjo Y Beral V Akiba S Possible protective effect of milk, meat and fish for cerebrovascular disease mortality in Japan J Epidemiol 1999 9 268 74 10510585 10.2188/jea.9.268

[25] Shimamoto T Iso H Iida M Komachi Y Epidemiology of cerebrovascular disease: stroke epidemic in Japan J Epidemiol 1996 6 43 47 10.2188/jea.6.3sup_43 8800273

[26] Hatano S Minowa M Omura T Stroke mortality and proportional expenditure on selected food items in Japanese communities Ann Clin Res 1984 16 Suppl 43 163 9 6535436

[27] Jarvis JK Miller GD Overcoming the barrier of lactose intolerance to reduce health disparities J Natl Med Assoc 2002 94 55 66 11853047 PMC2594135

[28] Takachi R Kimira M Uesugi S Kudo Y Ouchi K Watanabe S The effect of dietary and plasma fatty acids on platelet aggregation in senior generation of Japanese women Ann Clin Res 1984 16 Suppl 43 163 696535436

[29] Sone Y Tanida S Matsubara K Everyday breath hydrogen excretion profile in Japanese young female students Anthropol Appl Human Sci 2000 19 229 37 10.2114/jpa.19.229 11155352

[30] Mitsui T Shimaoka K Kanao Y Kondo T Colonic fermentation after ingestion of fructose-containing sports drink J Sports Med Phys Fitness 2000 41 121 3 11317159

[31] Kondo T Nakae Y Breath hydrogen and methane excretion produced by commercial beverages containing dietary fiber J Gastroenterol 1996 3 654 8 8887030 10.1007/BF02347612

[32] Suarez FL Springfield J Furne JK Lohrmann TT Kerr PS Levitt MD Gas production in human ingesting a soybean flour derived from beans naturally low in oligosaccharides Am J Clin Nutr 1999 69 135 9 9925135 10.1093/ajcn/69.1.135

[33] Cherbut C Inulin and oligofructose in the dietary fibre concept Br J Nutr 2002 87 S159 62 12088513 10.1079/BJNBJN2002532

